# From End to End: tRNA Editing at 5'- and 3'-Terminal Positions

**DOI:** 10.3390/ijms151223975

**Published:** 2014-12-22

**Authors:** Heike Betat, Yicheng Long, Jane E. Jackman, Mario Mörl

**Affiliations:** 1 Institute for Biochemistry, University of Leipzig, Brüderstraße 34, 04103 Leipzig, Germany; E-Mail: heike.betat@uni-leipzig.de; 2 Department of Chemistry and Biochemistry, Center for RNA Biology and Ohio State Biochemistry Program, the Ohio State University, Columbus, OH 43210, USA; E-Mails: long.918@osu.edu (Y.L.); jackman.14@osu.edu (J.E.J.)

**Keywords:** tRNA, editing, tRNA processing, tRNA maturation

## Abstract

During maturation, tRNA molecules undergo a series of individual processing steps, ranging from exo- and endonucleolytic trimming reactions at their 5'- and 3'-ends, specific base modifications and intron removal to the addition of the conserved 3'-terminal CCA sequence. Especially in mitochondria, this plethora of processing steps is completed by various editing events, where base identities at internal positions are changed and/or nucleotides at 5'- and 3'-ends are replaced or incorporated. In this review, we will focus predominantly on the latter reactions, where a growing number of cases indicate that these editing events represent a rather frequent and widespread phenomenon. While the mechanistic basis for 5'- and 3'-end editing differs dramatically, both reactions represent an absolute requirement for generating a functional tRNA. Current *in vivo* and *in vitro* model systems support a scenario in which these highly specific maturation reactions might have evolved out of ancient promiscuous RNA polymerization or quality control systems.

## 1. Introduction

**Figure 1:**
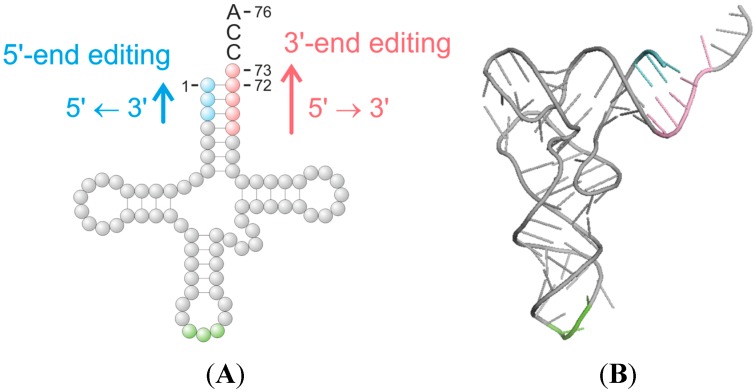
Editing events in the tRNA acceptor stem. (**A**) Cloverleaf secondary structure and (**B**) tertiary structure of a tRNA; the anticodon is indicated in green, positions of acceptor stem editing are shown in blue (5'-editing) and red (3'-editing). While the 3'-editing events occur in the conventional 5'-3'-polymerization direction of nucleic acids, the 5'-editing shows a highly unusual 3'-5'-direction, indicating that highly specialized polymerases are involved in this reaction. In contrast, the 3'-editing reaction can in principle be catalyzed by an RNA polymerase with conventional directionality, like the poly(A) polymerase, the CCA-adding enzyme, or the TRAMP complex (TRAMP: Trf4 or Trf5/Air2/Mtr4 polyadenylation; Trf: topoisomerase I-related function, poly(A) polymerase activity; Air: arginine methyltransferase-interacting RING finger, RNA binding protein; Mtr4: mRNA transport).

tRNAs play a central role as adapter molecules, linking the genetic information encoded in mRNA to the amino acid sequence of the encoded protein. To perform this critical function, tRNA molecules exhibit a characteristic structural organization that is conserved in all kingdoms of life. With a typical length of 70–85 nucleotides, the secondary structure resembles a cloverleaf (Figure 1A). Due to additional tertiary interactions and stacking of helices, tRNAs fold into the functional three-dimensional L-shaped form (Figure 1B). This structure allows tRNAs to perform their specific adapter role during protein synthesis, where one end of the L shape (the anticodon) specifically interacts with the corresponding codon in the mRNA, whereas the other end (the acceptor stem) is charged with the cognate amino acid that has to be delivered to the nascent protein (for review, see [1]).

Before tRNAs can fulfill this role in translation, the tRNA primary transcripts have to be extensively processed, as they are transcribed as precursor molecules with extra sequences at their 5'- and 3'-ends. In some cases, these transcripts even carry introns that have to be precisely removed [2,3]. During tRNA maturation, a series of individual processing steps occurs, which can include the removal of 5'-leaders and 3'-trailers by specific endo- and/or exonucleases, splicing of introns, and addition of the non-templated and universally conserved 3'-terminal CCA sequence, as needed to generate a functional full-length tRNA. Moreover, tRNA processing is not restricted to trimming and addition of these extra sequences, but also can include numerous specific base modification and editing events that are performed to generate the mature product. Although the distinction between tRNA editing and tRNA modification can be subject to some interpretation, for the purposes of this review, we will consider changes to the tRNA that result in the acquisition of non-canonical bases (such as the ubiquitous *N*-1-methylguanosine, pseudouridine or dihydouridine) to be modification events, while changes to the tRNA that result in acquisition of canonical bases (A, C, G or U) are considered to be tRNA editing events.

To date, more than 100 different nucleoside modifications have been identified in tRNA. Although the amount of modified nucleosides in tRNA varies substantially among different organisms, an average of up to 17% of the total residues in each tRNA is modified. Outside of a few well-studied cases, the precise function for many of these modifications remains largely unknown, and the abundant and conserved nature of these modifications suggests that they are important for ensuring correct tRNA structure and function [2,3,4].

In contrast to tRNA modification, the widespread process of tRNA editing is often needed to correct non-functional gene sequences at the RNA level, thus generating functional transcripts. In this context, the term tRNA editing describes a heterogeneous spectrum of reactions that alter RNA molecules post-transcriptionally by internal base substitutions, by nucleotide insertions and/or deletions, or even by the addition of 5'- or 3'-terminal sequences to truncated processing intermediates, all of which serve to generate a tRNA molecule that, in principle, could have been directly encoded at the DNA (gene) level [5,6].

The following facts illustrate how heterogeneous these editing events are. First of all, tRNA editing events are not restricted to a handful of organisms, but are frequently found in diverse eukaryotes, ranging from single cell organisms (*Trypanosomes*, *Acanthamoeba*, *Physarum*, *Spicellomyces*) to higher eukaryotes like plants (*Arabidopsis*) and animals (Metazoa) [5,6,7]. Interestingly, tRNA editing events are predominantly found in mitochondria and chloroplasts of these organisms, but rather rarely in the nucleus [8,9]. Moreover, although previously assumed to be restricted exclusively to eukaryotes, tRNA editing has been recently described in Archaea [10]. Secondly, the occurrence of tRNA editing relative to the timing of other processing events strongly differs for different examples of editing [7]. Whereas in some instances tRNA editing is a prerequisite for further processing steps [11,12], editing can also occur after 5'- and 3'-processing [13] or even after the 3'-terminal CCA addition [14]. Finally, the most prominent example of heterogeneity in tRNA editing is the fact that completely different types of reaction mechanisms and corresponding enzyme machinery have been associated with the various types of tRNA editing reactions described to date. Initially, 5'-end editing of tRNA transcripts was discovered in the mitochondrion of the protozoan *Acanthamoeba castellanii*, where 5'-mismatched nucleotides are removed and repaired by an editing enzyme that includes an unusual template-dependent polymerase that catalyzes nucleotide addition in the opposite direction (3'–5') as compared to canonical polymerases [5,13,15,16]. In the following years, a variety of additional tRNA editing reactions were discovered, all of which are mechanistically different and apparently involve distinct enzymes to catalyze the editing reactions [5].

tRNA editing events can be categorized in two general classes comprising either base substitution reactions or nucleotide insertion/deletion events [6]. In the case of substitution editing, these correspond to base deamination reactions, with deamination of cytidine to uridine (C to U substitution) as the most prevalent example [10,16,17,18], whereas the deamination of adenine to inosine (A to I editing) is a rather rare editing event, restricted to a few positions in the tRNA [3]. For editing events based on nucleotide insertions or deletions, a wide range of unusual template-dependent as well as template-independent editing events in the 5'- and 3'-parts of the tRNA acceptor stem are known so far [7,19] (Figure 1A). As with the base substitution events, these editing processes are likely to be critically important for generating functional tRNAs that can fulfill their task in translation and, consequently, to ensure the viability of the cell. In this review, we will describe the current knowledge on these highly fascinating specific nucleotide insertion and deletion reactions that occur within the tRNA acceptor stem.

## 2. tRNA Editing at 5'-Terminal Positions

### 2.1. Conventional 5'-End Processing

Nearly all precursor tRNAs are transcribed with 5'-leader and 3'-trailer sequences of variable length. The 5'-leader sequences are cleaved by the ubiquitous enzyme ribonuclease P (RNase P), with cleavage usually occurring before the N+1 nucleotide, generating a 5'-monophosphorylated end [20]. However, a few exceptions to this canonical cleavage position have been found. In one case involving histidine-decoding tRNA, RNase P cleaves to leave an additional 5'-end leader nucleotide, retaining a genomically-encoded guanosine residue (G-1) on tRNA^His^, which appears to be a requirement for tRNA^His^ recognition in Bacteria and organelles [21,22,23]. In another case, RNase P has been shown to miscleave certain precursor tRNAs with various frequency between the N+1 and N+2 nucleotide [24,25,26]. Unlike the case of tRNA^His^, this alternative cleavage reaction would not predictably generate a functional tRNA, and some still unidentified mechanisms may exist to repair these miscleaved tRNAs *in vivo*. Although removal of the 5'-leader sequence is a nearly universal process, so-called “leaderless” tRNAs were discovered in the archaeon *Nanoarchaeum equitans* [27]. In this species, a bioinformatics study suggested that the protein and RNA components of RNase P are not encoded in the genome, and instead, the precise placement of promoter sequences upstream of the tRNA 5'-end generates the correct 5'-start site [28]*.* Thus, cells exhibit multiple ways to generate tRNA with appropriate 5'-terminal sequences, and the process of tRNA 5'-editing can augment these processes to ensure a high quality tRNA pool.

### 2.2. Editing of the 5'-End Occurs on a Variety of Eukaryotic tRNAs

#### 2.2.1. Editing of Mismatches at 5'-Terminal Positions

**Figure 2:**
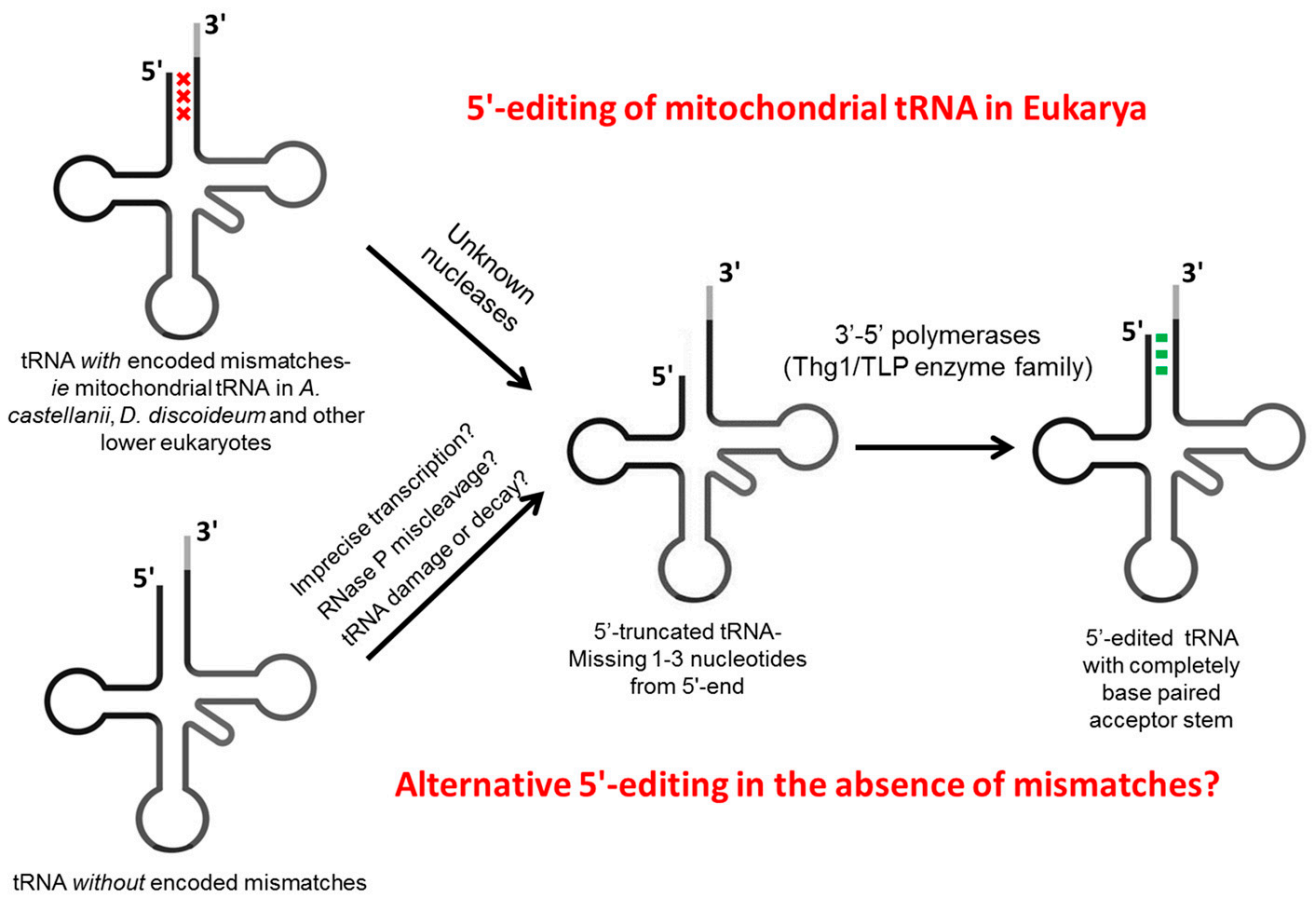
Known and predicted 5'-end editing pathways. 5'-end editing of mt-tRNA is associated with the presence of mismatched nucleotides at any of the first three positions of the tRNA acceptor stem (indicated by the red X). The top pathway shows the two-step editing process that repairs these mismatches, first by removal of the 5'-mismatched residues (by unknown enzymes) and next by repair of the resulting 5'-truncated tRNA by 3'–5'-polymerases known as TLPs (Thg1-like proteins; Thg1: tRNA^His^ guanylyltransferase), thus generating a fully base paired acceptor stem (Watson–Crick base pairs indicated by the green bars). The bottom pathway shows an alternative process that could also utilize similar 3'–5'-polymerases to edit tRNA in the absence of bona fide mismatches, such as in organisms where these types of mismatched nucleotides have not been identified in tRNA genes. In these cases, several possible pathways, as indicated, could conceivably generate 5'-truncated substrates to be repaired by Thg1/TLP orthologs found in these species.

tRNA editing was first discovered in the mitochondrion of *A. castellanii* [15,16]. In *A. castellanii*, according to the mitochondrial genome sequence, 12 out of the 15 mitochondrial-encoded tRNAs contain mismatches in one or more of the first three base pairs (1–72, 2–71 and 3–70) of the tRNA acceptor stem (Figure 2). These 12 tRNAs were sequenced, and it was found that the 5'-portion of each mismatch was edited by removal of the mismatched position and replacement with a nucleotide forming a correct Watson–Crick base pair, thus restoring the canonical base-paired stem [29]. Importantly, these editing events were not limited to pyrimidine to pyrimidine and purine to purine alteration, as occurs during the base substitution type editing events already known to occur in other RNAs (e.g., A to I or C to U editing) [3]. These results indicated that this tRNA editing event must utilize a distinct mechanism from the deamination type of editing that was commonly found in other lower eukaryotes. Interestingly, a tRNA^X^ that contains mismatches and has unknown functions *in vivo* was also edited, although its mature 3'-end was missing [29]. The large number of substrates that were observed to be edited indicated that the 5'-editing machinery likely exhibits a broad tRNA substrate specificity *in vivo*.

Similar 5'-editing events were subsequently documented in the mitochondria of several other lower eukaryotes, including members of Amoebozoa (*Physarum polycephalum*, *Dictyostelium discoideum* and *Polysphondylium pallidum*) and Fungi (*Spizellomyces punctatus*, *Harpotrichium* sp., *Monoblepharella* sp.) [30,31,32,33]. The editing process in these organisms appeared to be similar to what was observed in *A. castellanii*, and mismatches were restricted to the first three base pairs on the acceptor stem. In all of these cases, 5'-editing appeared to be coupled with other 5'-end processing events, implied by the fact that relatively few partially edited intermediates have been obtained from the sequencing experiments of these mitochondrial encoded tRNAs [13,30,33].

#### 2.2.2. Discovery of Components of the 5'-Editing Enzyme

Although tRNA 5'-editing was discovered in 1993, the editing machinery remained unknown for more than a decade. Unlike base substitution type RNA editing, (A to I editing and C to U editing), the nucleotide changes that occurred during 5'-editing resulted in substitution of any of the four nucleotides, which were apparently selected according to their ability to form canonical Watson–Crick base pairs in the acceptor stem. This observation suggested that the editing machinery utilized the sequences of the 3'-half of the acceptor stem as a template, implying that a nucleotidyl transferase activity might comprise part of the editing machinery.

Indeed, a novel nucleotide incorporation activity was later partially purified from the mitochondria of *A. castellanii* [13]. The nucleotide incorporation activity contained at least two components, first a 5'-exonuclease or endonuclease and second, a template-dependent 3'–5'-nucleotidyltransferase (Figure 2). The observation of this 3'–5'-polymerase activity was particularly unexpected, since this reaction involved synthesis in the opposite direction to all known DNA or RNA polymerases, and indeed enzymes capable of catalyzing this biochemical activity had not been identified at this time. This biochemical analysis also suggested that the nucleotidyl transferase component required an activated 5'-terminal residue, which could either be adenylylated (requiring ATP) or triphosphorylated, and that the activity generated 5'-triphosphorylated termini, suggesting that nucleotides were transferred as nucleoside triphosphates during the course of the reaction.

Although the requirement for a 3'–5'-nucleotidyl transferase was demonstrated, no enzyme capable of catalyzing this type of activity was known for another decade until an enzyme named tRNA^His^ guanylyltransferase (Thg1) was identified in the yeast *Saccharomyces cerevisiae*. In yeast and other eukaryotes, Thg1 is responsible for post-transcriptional incorporation of an essential G residue (G-1) at the 5'-end of tRNA^His^, and this nucleotide addition occurred in the 3'–5' direction [34,35]. Biochemical characterization revealed that, although addition of G-1 to tRNA^His^ does not generate a Watson–Crick base pair, the enzyme possesses a second template-dependent 3'–5'-polymerase activity that could be detected with tRNA^His^ variant substrates [36]. Moreover, Thg1 utilized an activated 5'-end, either generated by the ATP-dependent 5'-adenylylation reaction catalyzed by the enzyme itself, or by using transcripts containing 5'-triphosphorylated ends [35,36,37]. Finally, addition of each NTP generated 5'-triphosphorylated termini, which is very inefficiently hydrolyzed to a 5'-monophosphate if a Watson–Crick base pair is formed by the 3'–5'-addition reaction [38]. Thus, all of these biochemical properties of Thg1 fit the requirements of the nucleotidyl transferase component of the 5'-editing machinery. However, the inability of yeast Thg1 to catalyze nucleotide addition to 5'-truncated tRNA, such as would be generated during tRNA 5'-editing, suggested that Thg1 itself was not likely to be involved in the mitochondrial-type 5'-editing reactions [36].

Instead, the identification of the first enzymes likely to participate in 5'-editing of tRNA resulted from characterization of Thg1 orthologs from Bacteria and Archaea, which had been named Thg1-like proteins (TLPs) [39]. These TLPs exhibited key biochemical differences from the prototypical eukaryotic Thg1, in that they prefer to catalyze template-dependent 3'–5'-nucleotide addition, and importantly, also have the ability to repair 5'-truncated tRNAs in a templated manner [40,41,42]. Subsequently, TLPs were identified in the genomes of organisms where tRNA 5'-editing occurs. In the slime mold *Dictyostelium discoideum*, 10 out of the 18 mtDNA-encoded tRNAs are edited at the 5'-end, and one Thg1 and three TLPs are encoded in the nuclear genome [33,43]. *In vitro* biochemical assays indicated that two of these four enzymes (named DdiTLP3 and DdiTLP4) catalyze 5'-end repair of 5'-truncated mitochondrial tRNAs that correspond to the predicted substrates during the 5'-editing reaction, thus providing the first identification of enzymes that could be involved in the 5'-editing process [43]. Two TLPs (AcaTLP1 and AcaTLP2) were also identified in *A. castellanii*, and these enzymes also catalyze 5'-end repair of truncated editing substrates, inferring a role for either or both in the mitochondrial 5'-editing process that similarly occurs in this species [44]. The precise roles of each of these enzymes in the tRNA 5'-editing reaction awaits future *in vivo* characterization.

#### 2.2.3. Nucleases yet to Be Identified

Although progress has been made toward identification of the nucleotidyl transferase component of the 5'-editing enzyme, the identity of the nuclease(s) responsible for removing the 5'-mismatched nucleotides from the pre-tRNA transcript remains unknown (Figure 2). While the 5'-maturation enzyme RNase P is a possible candidate for participation in this reaction, given its ability to cleave 5'-nucleotides from pre-tRNA sequences, the requirement to efficiently cleave edited tRNAs within the aminoacyl-acceptor stem is at odds with the usual precision of RNase P cleavage at the +1 position, and its reliance on the integrity and length of the acceptor stem [45]. Alternatively, a 5'–3'-exonuclease or an endonuclease could be involved in the cleavage step, with or without prior 5'-end processing by RNase P. 5'–3'-exonucleases acting on tRNA have been identified, but enzymes such as Rat1 or Xrn1 that are involved in rapid tRNA decay (RTD) are generally highly processive and may be unlikely to participate in the limited nucleotide removal activity required for 5'-editing [46,47]. Noticeably, 5'–3'-nuclease activity is the reverse enzymatic reaction of the 3'–5'-nucleotidyl transferase chemistry, and in principle, it is possible that TLPs could act as the nuclease component as well. Finally, an endonuclease might recognize the 5'-terminal mismatches and cleave between the paired and unpaired nucleotides, thus directly generating the 5'-truncated end to be repaired by the TLP.

A challenge to identifying the nuclease component of the 5'-editing enzyme is that the 5'-end status of the pre-editing substrates is not precisely known. Due to the highly coupled nature of the 5'-editing and 5'-processing reactions, sequences of editing intermediates are difficult to obtain in sufficient numbers to determine the precise form of the edited tRNA after the nuclease activity and before the repair step of the reaction has occurred. In a few cases, partially edited tRNA molecules have been observed through tRNA sequencing reactions, and some of these tRNAs appear to have undergone incomplete removal of the mismatched 5'-nucleotides, suggesting that the nuclease activity may occur in multiple steps (although this does not exclude the involvement of either endo- or exonucleolytic activities, since either could be consistent with this result) [30,33]. Inherent biases of the ligation-dependent techniques typically used to sequence the isolated tRNA may also result in incorrect reconstruction of the editing reaction, and the precise order and composition of events that makes up the editing reaction remains to be determined. Even the possibility of more extensive nucleolytic activity to remove additional nucleotides beyond the 5'-mismatched residues cannot be ruled out, since repair of this type of intermediate would simply regenerate Watson–Crick base paired nucleotides that could not be distinguished from the pre-tRNA transcripts. Finally, as many of the mitochondrial tRNAs that undergo 5'-editing are clustered in polycistronic transcripts, it is also possible that the 3'-processing machinery of an upstream tRNA could directly provide a downstream tRNA substrate for the editing machinery.

#### 2.2.4. G:U/U:G Base Pair Editing: Diversity among Organisms

G:U/U:G base pairs are frequently observed in stems of tRNA, even serving as identity elements recognized by aminoacyl synthetases, and thus are not typically considered to be mismatches that would need to be repaired in order to generate a functional tRNA [48,49,50]. Interestingly, though, several examples of G:U/U:G base pairs that are replaced by canonical Watson–Crick pairs have been documented [30,33], and whether these G:U/U:G base pairs are edited by the 5'-editing reaction apparently differs greatly between organisms. For example, in *P. pallidum* G:U/U:G pairs within the first three base pairs of a tRNA are rarely edited, while in *D. discoideum* G:U/U:G base pairs in similar context are frequently edited [33]. It is currently unclear what determines whether G:U/U:G base pairs are subject to 5'-editing, although contributions from either the nuclease or nucleotidyl transferase steps of the editing reaction are possible. The as of yet unidentified nucleases from different organisms could exhibit different substrate specificities in terms of G:U/U:G pairs, thus resulting in either retention or removal of the G:U/U:G pair during this first step of the 5'-editing reaction. An organism-specific contribution to this diversity could also be associated with the second step of 5'-editing, where editing-related TLPs could exhibit distinct substrate preferences regarding the ability to accommodate G:U/U:G base pairs during 3'–5'-repair reactions.

Initial observations of G:U/U:G base pair editing suggested that these types of base pairs were only recognized for editing if they were present in a tRNA along with other types of non-Watson–Crick mismatches. However, in *D. discoideum*, two edited tRNA were recently identified, where G:U/U:G pairs at the 1:72 position of the tRNA are edited in the absence of any other bona fide mismatches [33]. Interestingly, in this case, three other *D. discoideum* tRNA species that contain G:U/U:G base pairs, but no other mismatches, at internal positions in the aminoacyl-acceptor stem are not edited, and thus the G:U base pairs remain unchanged in these tRNA. For one of these tRNAs, tRNA^Ala^, the G3:U70 base pair actually serves as an identity element for aminoacylation, and therefore the absence of editing is biologically important for this substrate. Nonetheless, the observation of an example of editing in the absence of any other mismatches supports a hypothesis that the tRNA editing machinery may have been derived from a constitutive removal/repair activity that could also act as a 5'-end surveillance machinery for tRNA quality control. This hypothesis is also consistent with the earlier observation that the partially purified editing activity from *A. castellanii* incorporated nucleotides into mature tRNA^Tyr^ derived from *E. coli*, which lacks any obvious mismatches [13]. A complete understanding of the 5'-editing reaction will require further delineation of the rules that govern the selection of which tRNA species and base pairs are subject to this editing process.

#### 2.2.5. Alternative Roles for 5'-End Editing Outside of Mitochondria?

TLP enzymes were originally identified in Bacteria and Archaea, where the annotated tRNA species do not encode 5'-mismatches, and thus the mitochondrial-type 5'-editing reaction does not occur. Yet, bacterial and archaeal TLPs catalyze the repair of 5'-truncated tRNAs *in vitro*, and the physiological substrates for these enzymes have not been identified [42]. One possibility is that these TLPs may act as part of a tRNA surveillance pathway and repair 5'-misprocessed tRNA (Figure 2). For example, characteristic RNase P miscleavage events with bacterial tRNA^Gln^ generate a 5'-end that lacks the C+1 nucleotide [26]. Although in this case, the misprocessed tRNA comprises only a minor fraction (~25%) of the tRNA^Gln^, a pathway to repair this pool of presumably non-functional transcripts may be particularly important under conditions of rapid growth or cellular stress. In this way, the 5'-end repair pathways may be similar to the role of 3'-end repair by the CCA-adding enzyme in some bacteria, which is not strictly essential to the cell [51,52]. Other possible substrates for TLPs, perhaps generated by RNase P, 5'–3'-exonucleases or variability in transcription start site remain to be investigated, and TLP enzymes could even conceivably play a role in sensing and repairing other types of base damage to tRNA, which may indicate broader connections between RNA repair and editing activities than are currently understood. Although all the types of activities described above could readily be classified as tRNA quality control, rather than tRNA editing *per se*, the fact that these misprocessing events could be corrected at the post-transcriptional level merits consideration along with the more traditional editing pathways. It is notable that if bacterial or archaeal 5'-editing reactions such as those described above occur, these would be the first examples of 5'-end editing observed outside of eukaryotic organelles.

#### 2.2.6. C to U Editing at the 5'-End

Although base substitution-type editing reactions are not the primary focus of this review, a few C to U editing events have been found at positions close to the 5'-end of several tRNAs, particularly in plant mitochondria, and raise questions about possible connections to the 5'-end editing reactions discussed above. In the mitochondrion of dicots, a C4-A69 mismatch in tRNA^Phe^_GAA_ is corrected to U4-A69 Watson–Crick base pair, presumably restoring the integrity and stability of the acceptor stem [53,54]. C to U editing has also been observed at position 6 and 8 on tRNAs, where canonical base pairs or tertiary interactions were restored to maintain the overall tRNA structure [10,12]. Presumably, these C to U changes are introduced by deaminases like the cytidine deaminase homolog (CDAT8) that acts on C8 in *Methanocryptus kandlerii* [10]. In this reaction, the base portion of the nucleotide is modified without breaking any phosphodiester bonds, and the requirement for C to U editing as a prerequisite for precursor processing in plant mitochondria suggests a similar deaminase-catalyzed mechanism occurring on the unprocessed precursor transcript. However, the existence of other C to U editing reactions that introduce these changes in the acceptor stem from similar removal and repair mechanisms to those involving TLPs cannot be strictly ruled out.

### 2.3. Specialized Editing at the 5'-End of tRNA^His^

#### 2.3.1. G-1 Editing of tRNA^His^ in Eukaryotes

**Figure 3:**
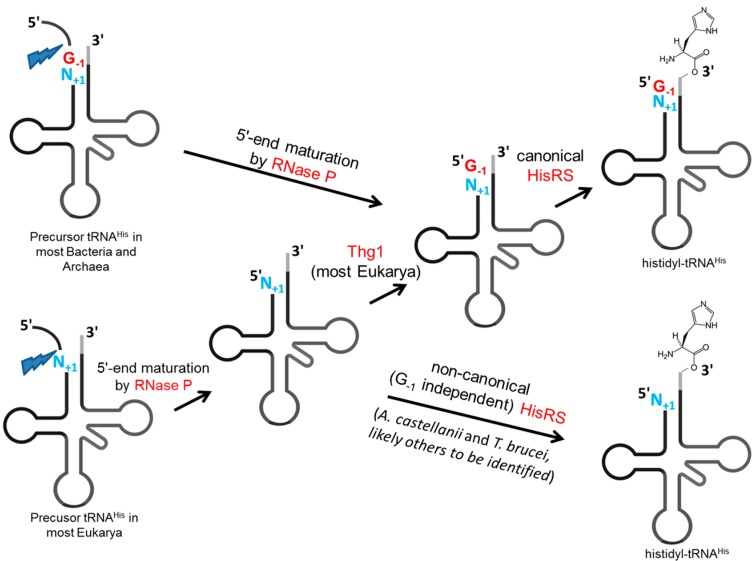
A specialized 5'-editing pathway that acts on tRNA^His^. In Bacteria and Archaea, tRNA^His^ species are almost always encoded with an additional 5'-G nucleotide that is retained during 5'-end maturation by RNase P (indicated by the blue lightning bolt), ensuring the presence of the 5'-G-1 residue that is required to recognize the tRNA^His^ by its canonical Histidyl-tRNA synthetase (HisRS). However, in most eukaryotes, the pre-tRNA^His^ does not encode the additional G-1 and eukaryotic Thg1 enzymes catalyze a specialized 5'-end editing reaction to post-transcriptionally add G-1 to the tRNA and thus ensure proper recognition. Recently, some eukaryotes have been identified that lack a bona fide Thg1 ortholog, and in these cases, the lack of the 5'-edited tRNA^His^ is accommodated by the presence of a non-canonical HisRS that is capable of efficiently aminoacylating the tRNA even in the absence of G-1. The G-1 nucleotide is indicated in red, and the N+1 nucleotide that represents the normal 5'-end for all other tRNA species is highlighted in blue.

A special 5'-editing event occurs on cytoplasmic tRNA^His^ in eukaryotes. Compared to other tRNA species, tRNA^His^ from almost all organisms contains an additional 5'-guanylate residue (G-1), which serves as an important identity element for histidyl-tRNA synthetase (HisRS) [55,56,57,58]. In bacteria, this G-1 residue is genomically encoded, and RNase P cleaves before the G-1 residue to generate the mature 5'-end containing an additional G-1:C73 base pair (base positions according to the standard numbering of tRNAs), which is important for correct recognition of tRNA^His^, with the C73 residue playing a particularly critical role for correct aminoacylation *in vivo* [21,22,23,59] (Figure 3). In eukaryotes, the discriminator residue is an A73, and although artificial substitution of C73 into *S. cerevisiae* tRNA^His^ improves aminoacylation detectably *in vivo*, the presence of C73 alone in the absence of G-1 is not sufficient to support aminoacylation in otherwise wild-type cells [60]. However, in contrast to bacteria, most eukaryotes lack the genomically-encoded G-1, and hence a special process is required to generate a G-1-containing tRNA^His^ [61] (Figure 3). Although here again, the distinction between tRNA modification and tRNA editing is somewhat blurred for this G-1 addition reaction, the process involves post-transcriptional addition of a standard RNA nucleotide that differs from the encoded nucleotide, and thus can be considered a tRNA editing event for the purposes of this review.

The canonical pathway for G-1 addition has been well characterized in *S. cerevisiae* and also involves two steps. First, RNase P acts as an endonuclease to cleave between the N−1 and N+1 nucleotides, removing the incorrect N−1 residue along with the 5'-leader sequence. Second, Thg1 utilizes its 3'–5'-nucleotidyltransferase activity to incorporate the correct G-1 residue to the 5'-end, generating a G-1 containing tRNA^His^ [35] (Figure 3). This special G-1 editing process is strikingly similar to the mitochondrial 5'-editing process described above. While it is unclear whether RNase P is also responsible for the cleavage step of the mitochondrial 5'-editing, the second steps of both editing reactions share nucleotidyl transferases from the same 3'–5'-polymerase superfamily [39]. Noticeably, the mechanism for selecting the correct nucleotide to incorporate differs between the two editing processes, since during G-1 editing on tRNA^His^, Thg1 enzymes strictly incorporate a G-1 residue across an A73 residue at the 3'-end, thus creating a non-Watson–Crick base paired 5'-end. However, in the mitochondrial 5'-editing process, TLP enzymes exhibit a strong preference to incorporate complementary nucleotides to create Watson–Crick base pairs. Elucidating the molecular basis of this nucleotide specificity would lead to better understanding of the two editing events and the evolutionary process of the 5'-editing machinery.

#### 2.3.2. Life without G-1

Although G-1 is a nearly universal hallmark of tRNA^His^ in all three domains of life, tRNA^His^ lacking the G-1 residue has been found in a group of α–proteo Bacteria including the *Rhizobiales*, *Rhodobacterales*, *Caulobacterales*, *Parvularculales*, and *Pelagibacter* [62]. These organisms do not encode the G-1 residue, as is observed with all other bacteria, and G-1 cannot be added post-transcriptionally due to the absence of any encoded Thg1 enzymes in these species. The loss of G-1 on tRNA^His^ is complemented by alterations to highly conserved residues of their corresponding HisRS, thus adapting to the lack of this important identity element for proper tRNA recognition [63,64].

More recently, it was demonstrated that the G-1 residue can also be missing in eukaryotes. Investigation of several eukaryotes lacking obvious Thg1 homologs revealed that cytoplasmic tRNA^His^ isolated from *A. castellanii* and *Trypanosoma brucei* lack the G-1 residue [44]. Although two TLPs exist in *A. castellanii*, neither of them is capable of catalyzing G-1 addition on the cytoplasmic tRNA^His^ *in vitro*, which is consistent with the general inability of TLPs to catalyze the non-Watson–Crick G-1 addition, and *T. brucei* does not encode any gene that is a member of the Thg1/TLP superfamily [39,44]. Similar to the situation in α-proteobacteria, the cytoplasmic HisRS enzymes from *A. castellanii* and *T. brucei* efficiently aminoacylate tRNA^His^ in the absence of the universal G-1 residue, but the lack of sequence similarity between these enzymes and their bacterial counterparts suggests an alternative mechanism by which this non-canonical substrate specificity is achieved. The evolutionary significance of loss of the G-1 identity element is unclear, but the scattered distribution of organisms that lack an identifiable Thg1 homolog among eukaryotes suggests that additional examples of this type of G-1-independent tRNA^His^ recognition may yet be identified.

## 3. The Heterogeneity of Editing Mechanisms at the tRNA 3'-End

In all kingdoms of life, the integrity of the tRNA acceptor stem 3'-end is an essential prerequisite for a tRNA to participate in translation. Hence, in addition to ensuring the presence of correct nucleotides at the tRNA 5'-end, editing events located in the 3'-region are equally important. Research in the last 15 years revealed that, especially in metazoan mitochondria, many tRNA molecules are transcribed with mismatches or missing nucleotides at the tRNA 3'-end. These mismatches/deletions are the result of deviations in gene sequences or of overlapping regions between two adjacent tRNA genes encoded on the same strand of DNA. Hence, sequence corrections at the 3'-end of metazoan mitochondrial tRNAs are rather frequently observed. Although none of the responsible enzymatic activities have been identified yet, a series of template-dependent as well as -independent editing reactions has been proposed to restore correct base pairing in the tRNA acceptor stems [65,66,67,68,69,70].

### 3.1. Template-Independent Mechanisms of tRNA Editing at tRNA 3'-Ends

Mitochondrial genomes of metazoans are highly condensed and encode only a minimal set of tRNAs essential for a functional protein biosynthesis [71]. As a consequence of this condensation, tRNA genes overlapping by one to six positions with neighboring tRNA genes are quite common in these mitochondrial genomes, with a high prevalence for overlaps between tRNA^Tyr^ and tRNA^Cys^ or tRNA^Ser^_CGU_ and tRNA^Leu^. In most situations, the involved tRNA genes are encoded on the same strand and share a stretch of up to six nucleotides. As the overlapping tRNAs are transcribed as a single polycistronic precursor transcript, only one tRNA can be released as a complete molecule, while the other tRNA lacks the overlapping region and is therefore truncated. Alternative processing events that produce the complete upstream tRNA from one precursor and the downstream tRNA from another separate transcript, could be excluded [67]. Instead, *in vivo* as well as *in vitro* data indicate that exclusively the downstream located tRNA is released as a complete transcript, while the upstream tRNA lacks the overlapping part and is therefore truncated at its 3'-end. These missing positions are then restored in an editing reaction, and the subsequent addition of the CCA terminus finally converts the transcript into a mature and functional tRNA (for review, see [7]).

In the case of the Japanese land snail *Euhadra herklotsi* [68] and the squid *Loligo bleekeri* [72], these tRNA 3'-editing reactions lead to the addition of short stretches of A residues. In these organisms, most of the overlapping tRNAs carry stretches of U residues in the 5'-part of the acceptor stem that could function as a template for the addition of A residues in the 3'-part, restoring U–A base pairs. However, in the snail mitochondrial tRNA^Tyr^, representing one of three edited tRNA transcripts, the editing event generates an A1–A72 mismatch (base numbering according to [73]; Figure 1A) at the first base pair position of the acceptor stem. Furthermore, the unpaired discriminator position 73 (that cannot be templated due to the absence of a corresponding base at the tRNA 5'-end once it has been processed) was also restored by an A residue [68]. A similar situation was found in the same tRNA in squid mitochondria. Here, the first acceptor stem base pair U1–A72 as well as the discriminator A73 were restored, the latter again in a template-independent way [72]. In both cases—mismatch generation and discriminator addition—a template-independent poly(A) polymerase-like activity was proposed to be responsible for this type of tRNA editing reaction [68,72], similar to the activity that completes UAA stop codons in some mitochondrial mRNA transcripts [74,75]. A surplus of the added adenosine residues seems to be removed by polynucleotide phosphorylase (PNPase; [74]) or 2'-phosphodiesterase (PDE12; [76]). In a third example, Yokobori and Pääbo demonstrated that in the chicken *Gallus gallus*, the addition of a single A residue restores the discriminator position at the mitochondrial tRNA^Tyr^, prior to CCA addition [69]. Again, a polyadenylation-like mechanism for this type of editing was discussed. However, even after almost 20 years, the enzymes responsible for these editing events are not identified. While the events described above represent exclusively A additions, 3'-end editing in the mitochondrial tRNA^Ser^_GCU_ in the platypus *Ornithorhynchus anatinus* involves also C incorporations. Here, the acceptor stem is restored by the addition of three C residues, including an A2–C71 mismatch. At the discriminator position 73, however, a single A is incorporated [70]. Accordingly, the authors suggest that the involved editing mechanism must differ from the above mentioned polyadenylation event. Yet, due to the mismatch and the unpaired position 73, this unidentified activity again seems to be template-independent.

Another quite well characterized editing system is found in human mitochondria, where the genes for tRNA^Tyr^ and tRNA^Cys^ overlap by a single adenosine residue [67]. As described for the other cases indicated above, *in vitro* as well as *in vivo* studies showed that the upstream tRNA^Tyr^ is released as a truncated molecule, where the missing adenosine, representing the discriminator position, is restored. Subsequently, the edited tRNA is completed by the addition of the terminal CCA sequence. Interestingly, even further 3'-terminally truncated tRNAs were accepted as substrates for editing by a mitochondrial protein extract, leading to the incorporation of C and A residues [77]. Since mutations in the corresponding 5'-part of the acceptor stem did not affect the incorporation of these nucleotides, it was concluded that this editing reaction occurs in a template-independent way. Due to the nature of the incorporated nucleotides, it was assumed that tRNA nucleotidyltransferase (CCA-adding enzyme) might be involved in this reaction. However, experiments with a set of recombinant CCA-adding enzymes from different organisms has shown that these enzymes are not able to restore these truncated 3'-parts of tRNAs [67,78]. Hence again, the involved editing activity remains unclear.

Although the nature of the corresponding editing activities working at the tRNA 3'-end in the different organisms is not known, Reichert and coworkers proposed a model to explain how a template-independent editing activity might be able to restore different base pairs in the tRNA acceptor stem as well as the unpaired discriminator base [77]. According to this model, a template-independent editing activity inserts the missing nucleotides in a random manner. Then, only correctly restored tRNAs can be recognized by the corresponding aminoacyl tRNA synthetase and charged with the cognate amino acid. As a consequence, the charged tRNA is protected against exonucleolytic degradation. In the case of nucleotide misincorporations, the tRNAs are not aminoacylated, and their 3'-ends are not protected. Exonucleases can remove the misincorporations and the editing activity has a second chance to incorporate the correct nucleotides, resulting in a functional and aminoacylated tRNA protected from further degradation [77].

### 3.2. Template-Dependent Editing Reactions at tRNA 3'-Ends

An extreme case of mitochondrial tRNA 3'-editing was observed in the centipede *Lithobius forficatus* [65]. In contrast to the other editing events described above, where only very few tRNAs are subjected to editing, 21 of 22 tRNAs have to be completed by 3'-terminal nucleotide additions. These editing events not only affect truncated tRNAs resulting from overlapping tRNA genes; rather, editing is also required to remove as many as five encoded mismatches in the acceptor stems. In contrast to the above mentioned 3'-editing mechanisms that add only A and C residues in order to restore the tRNA structure, all four nucleotides are utilized in the editing reactions of this centipede. Interestingly, the edited transcripts show perfect base pairing in the restored acceptor stems, indicating that the 5'-end of the acceptor stem might represent a template for nucleotide addition at the tRNA 3-end. Consequently, this novel kind of tRNA editing would require an RNA-dependent RNA polymerase, as it is encoded in the mitochondrial genome of *Arabidopsis thaliana* and a virus-like RNA in the fungus *Ophiostoma novo-ulmi* [79]. However, as the discriminator position 73 is unpaired, there is no template for this nucleotide addition. Hence, it was assumed that for this position, a template-independent activity is required. In the jakobid *Seculamonas ecuadoriensis*, similar editing reactions in the mitochondrial tRNAs for serine (GGA) and glutamate have been described, leading to the preferential addition of C and A residues to replace the mismatched base at the tRNA 3'-end [66]. While a template-dependent incorporation similar to the *L. forficatus* situation cannot be ruled out, Leigh and Lang suggested that the jakobid tRNA editing is mechanistically different. With a strong bias for C and A addition restricted to the terminal three nucleotide positions 71–73, the authors favor a CCA-adding enzyme-like editing activity [66].

Taken together, tRNA 3'-end editing reactions are found very frequently in the mitochondria of metazoans. However, neither the molecular mechanisms underlying the individual reactions nor the responsible enzymatic activities have been identified so far, and further experimental work is needed to identify the nucleotidyl transferases responsible for these unusual and fascinating maturation and repair events.

## 4. *Saccharomyces cerevisiae* as a Model Organism: Evolution of tRNA Editing

### 4.1. The RNA Surveillance Complex TRAMP4 Shows tRNA 3'-Editing Activity in Yeast

Besides the unknown enzymatic activities that catalyze these numerous examples of tRNA editing, the evolutionary origin of the editing events represent a mystery as well. Given the bacterial origin of mitochondria [80], it is not clear why these events are restricted to these organelles but are not found in prokaryotic systems. To investigate whether Bacteria carry a tRNA maturation machinery that is compatible with overlapping tRNA precursors, Schuster and coworkers introduced a bicistronic tRNA^Tyr^/tRNA^Cys^ transcript, overlapping for one position, into *E. coli* [78]. Interestingly, it turned out that the prokaryotic tRNA processing machinery of *E. coli* is not compatible with the need to convert overlapping tRNAs into two mature and functional transcripts, as the endo- and exonucleolytic tRNA processing machinery [81,82,83] simply degraded the downstream located tRNA during processing the upstream tRNA, which was released with a complete 3'-end. Based on these results, it seems that the prokaryotic endosymbiont that evolved into modern mitochondria adopted the tRNA maturation pathway of the host cell that is compatible with the processing of precursor transcripts carrying overlapping tRNA copies, as it consists exclusively of endonucleases RNase P and tRNase Z that precisely cleave at the tRNA 5'- and 3'-ends [78,84]. Due to this incompatible prokaryotic tRNA processing mechanism, it is very likely that similar editing events did not evolve in prokaryotes and represent a specific feature of eukaryotic cells. Nevertheless, it is possible that the repair function of the bacterial CCA-adding enzyme or the activity of other nucleotidyltransferases are involved in the restoration of hydrolytically truncated tRNA ends, supporting the idea that tRNA editing and repair might be evolutionarily closely related (see Section 4.2.). This is in agreement with the finding that the nuclear/cytosolic tRNA processing system of *Saccharomyces cerevisiae* (a eukaryote that also does not carry tRNA gene overlaps) is nonetheless able to process such overlapping transcripts. Here, the same overlapping bicistronic tRNA^Tyr^/tRNA^Cys^ transcript was processed to release a complete downstream tRNA^Cys^ (carrying the overlapping position) and a corresponding 3'-terminally truncated tRNA^Tyr^, which was subsequently completed by the addition of the missing nucleotide. Obviously, *S. cerevisiae* carries the complete enzymatic equipment to process precursor overlaps and to edit 3'-truncated tRNAs, although its nuclear and mitochondrial genomes do not encode such overlapping tRNA genes. Hence, it is possible that yeast might be on its way to evolve tRNA editing [78].

In a subsequent study, the corresponding tRNA editing activity in yeast was identified [85]. An initial screen of the yeast genome for genes encoding enzymes with nucleotidyltransferase domains as possible editing enzymes revealed 119 candidates. As the editing event is obviously template-independent, standard DNA and RNA polymerases were excluded, leaving a set of four activities. According to earlier studies [67,78], the CCA-adding enzyme as well as the poly(A) polymerase could be ruled out, leaving the noncanonical poly(A) polymerases Trf4p and Trf5p. Both enzymes are part of two isoforms of the TRAMP (Trf4 or Trf5/Air2/Mtr4 polyadenlyation) complex involved in RNA quality control, where it labels non-functional transcripts with poly(A) tails as degradation tags [86,87,88]. This multi-protein TRAMP complex consists of the RNA helicase Mtr4 (mRNA transport), one of the RNA-binding zinc knuckle proteins Air1 or Air2 Arginine methyltransferase-interacting RING finger protein) and either Trf4 (TRAMP4) or Trf5 (TRAMP5) respectively. The analysis of the tRNA editing efficiencies in knock-out strains for genes *trf4* as well as *trf5* showed that Trf4p is the responsible activity that restores the missing nucleotide at the tRNA 3'-end. Furthermore, Air2p is equally important for this reaction. As Trf4p and Air2p are typical components of TRAMP4, it was concluded that this complex is catalyzing the tRNA editing reaction in yeast [85].

### 4.2. About the Evolution of tRNA 3'-End Editing

The finding that a polymerase involved in RNA quality control is able to efficiently edit truncated tRNA transcripts is the first experimental support of the general hypothesis that RNA editing evolved out of a pre-existing enzymatic activity that was promiscuous enough to tolerate new substrates for nucleotide additions [78,89,90,91,92]. In this scenario, a first event is the occurrence of a mutation in a gene, leading to a non-functional transcript like a truncated tRNA. In many cases, such an event is lethal for the cell, resulting in a rapid clearance when under selection. However, if the cell carries a nucleotide-adding activity (a nucleotidyl transferase) that accepts the truncated tRNA and adds some nucleotides that complete the transcript, the tRNA could be converted into a functional molecule. As a consequence, the mutation will not be detrimental for the cell, and the reaction will be evolutionary fixated, as it allows the survival of the organism.

**Figure 4:**
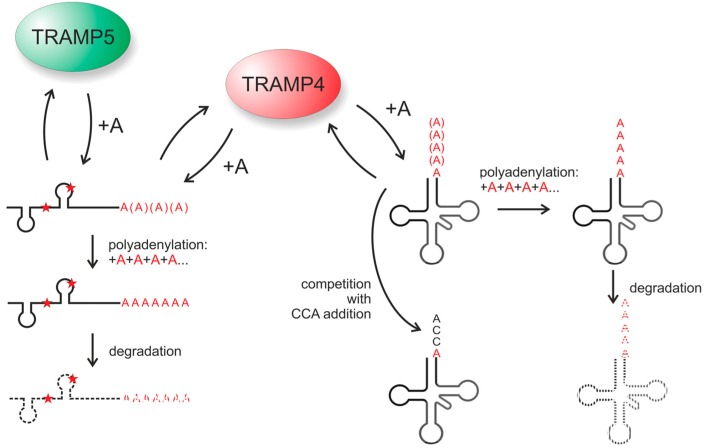
The TRAMP4 complex as an editing enzyme. Both TRAMP4 and TRAMP5 are important poly(A) polymerases involved in RNA quality control. **Left**: By the distributive addition of short poly(A) tails to a misfolded transcript (indicated by the red asterisks), these RNAs are tagged for subsequent degradation; **Right**: TRAMP4 also adds a restricted number of A residues to 3'-terminally truncated tRNA transcripts. If more A residues are added, leading to poly(A) tails, the tRNA is also labeled for subsequent degradation. However, as TRAMP4 is also a distributive polymerase that leaves the RNA substrate after the addition of a single A residue, the CCA-adding enzyme has a chance to bind to the restored tRNA, adding the CCA-terminus. In this case, TRAMP4 does not add a poly(A) tail, and the tRNA that now carries a CCA-end is functional and can participate in translation. Hence, the single A addition catalyzed by TRAMP4 corresponds to a true tRNA 3'-editing event. It is very likely that during evolution, similar RNA polymerases involved in RNA surveillance were recruited as editing enzymes.

Although the observed tRNA 3'-end editing occurs in the nucleus and/or cytosol of yeast, but not in the mitochondria, the TRAMP4 complex fulfills all the requirements for such a hypothetical editing activity. First, its function in RNA quality surveillance requires that it has a rather broad substrate spectrum and accepts most—if not all—damaged RNA transcripts of the cell for polyadenylation (Figure 4). Consequently, it also tolerates truncated tRNA transcripts for nucleotide incorporation. Furthermore, the editing reaction requires the addition of individual A residues, but not the synthesis of long poly(A) tails, where subsequent trimming reaction would be required. Although such trimming events might be involved in some tRNA editing events, as indicated above, in the case where only a single A residue has to be added, a distributive nucleotide incorporating activity would be more efficient. Indeed, the TRAMP complex is not processive, but adds A residues in a distributive manner, repeatedly binding and releasing the RNA substrate after the addition of a single nucleotide [84]. After dissociation from the TRAMP complex, the edited tRNA (carrying the added A residue at the 3'-end) would then be accepted by the CCA-adding enzyme and converted into a mature and functional transcript (Figure 4). This scenario of a promiscuous activity that is recruited for new functions is surely not restricted to tRNA editing, but is very likely to represent a general strategy in evolution. The identification of further editing activities will shed more light into this fascinating and essential maturation events that are mechanistically so diverse.

## 5. Conclusions

5'- and 3'-editing are widespread phenomena known to affect a variety of tRNAs in the context of a significant number of eukaryotic organisms. A comparison of the two editing processes is summarized in Table 1. Although there is a basic understanding of the types of editing reactions that can occur at either end of the tRNA, there remain many outstanding questions about the interplay and coordination of these two reactions with other tRNA processing events, as described below.

**Table 1:** Comparison of 5'- and 3'-editing reactions.

		5'-Editing	3'-Editing
Similarities	Locations	mitochondria and chloroplast in eukaryotes	
	Mechanism	nucleotide removal and transfer	
	Usage	restore base pairs, make functional tRNAs	
Differences	Enzyme	3'-5'-polymerases (Thg1-like proteins, or TLPs) and unknown enzymes	unknown enzymes, possibly similar to TRAMP complex
	Nucleotidyl transfer reaction	3'-5': template-dependent	5'-3': both template-independent and template-dependent
	Substrates	mismatch-containing tRNAs	Overlapping tRNAs and some mismatch-containing tRNAs

### 5.1. What Determines Which Half to Edit?

5'- and 3'-editing both occur on tRNAs containing mismatches in the acceptor stem, and both editing processes include removal of the mismatched nucleotides and addition of the correct ones to restore the integrity of the acceptor stem. Although both editing events can correct mismatches to canonical base pairs, in each case there is a clearly observed pattern of removal and repair of either the 5'- or 3'-end nucleotides, depending on the type of editing reaction that should occur. However, the answer to the question of what determines whether the 5'- or the 3'-end gets edited is difficult to answer in light of the as of yet unknown nature of the editing enzyme machinery that carries out the majority of these reactions. Interestingly, the 3'–5'-polymerases associated with the 5'-editing machinery are not found in mitochondria of all eukaryotes. For example, in *L. forficatus* where 21 out of 22 mitochondrial encoded tRNAs contain mismatches, 3'–5'-polymerases of the Thg1/TLP superfamily are not identified by BLAST search, leading to the inference that only 3'-editing can be utilized to correct those mismatches, and that perhaps the presence of a specific editing machinery dictates the selection of the portion of the tRNA subjected to editing. So far, 5'- and 3'-editing have not been identified in the same organism, but it would be interesting to study how mismatches are corrected in the presence of both editing systems, if these situations arise.

### 5.2. Can 5'-Editing Be Used to Process Overlapping tRNAs?

So far, 3'-editing, but not 5'-editing, is the predominant pathway to process overlapping tRNAs, where these examples have been investigated. In these cases, an overlapping transcript is always cleaved to yield a 3'-truncated upstream tRNA and a 5'-full-length downstream tRNA. Mechanistically, however, 5'-editing would also be capable of processing these overlapping transcripts, if the maturation machinery released a full-length upstream tRNA and a 5'-truncated downstream molecule. It is challenging to study whether 5'-editing is used to process overlapping tRNAs due to the apparent cooperativity of the 5'-processing and 5'-editing, making it difficult to observe processing intermediates using conventional sequencing approaches.

An opportunity to clarify this situation is found in the mitochondria of *D. discoideum*, where two mt-tRNA (tRNA^Ile^_GAU(1)_ and tRNA^Ile^_GAU(2)_) share one overlapping nucleotide such that A1 of the downstream tRNA (tRNA^Ile^_GAU(2)_) can also be considered as A73 of the upstream tRNA (tRNA^Ile^_GAU(1)_). The downstream tRNA contains two mismatches (A1–G72 and A2–A71) in its acceptor stem, and it has been shown that both A1 and A2 are edited to C1 and U2 to restore the Watson–Crick base pairs [33]. It is difficult to predict how the mitochondrial RNase P, which has not been studied in *D. discoideum*, or other nucleases cleave this polycistronic transcript. If the overlapped A is kept as the A1 residue in the downstream tRNA, as in other examples of overlapping tRNAs, the upstream tRNA would be missing the A73 discriminator base, and thus require 3'-editing machinery to add this residue, which would lead to the first discovery of 3'-editing and 5'-editing in the same organism. If the overlapping A is kept as the A73 residue of the upstream tRNA, the cleavage would generate a 5'-truncated downstream tRNA, which has never been observed in processing of overlapping tRNAs, and it would be interesting to identify the nucleases responsible for this cleavage.

### 5.3. Editing Machinery and tRNA Surveillance

Although there are no obvious editing substrates in *S. cerevisiae*, 3'-editing machinery (in the form of the TRAMP complex) nonetheless exists and is capable of processing overlapping tRNAs. However, because the TRAMP complex also acts in tRNA surveillance, the presence of overlapping or mismatched nucleotides may not necessarily be required for tRNA editing to occur, and these types of reactions could yet play an important role in tRNA quality control. Likewise, for 5'-editing, the 3'–5'-polymerases capable of repairing 5'-truncated tRNA are also found in a significant number of organisms where no mismatch-containing tRNA are identifiable in the genome, indicating a similar potential function for these enzymes in tRNA quality control pathways. Since the 5'- and 3'-termini are subject to rapid tRNA decay and 3'-end decay pathways, 5'- and/or 3'-editing could play roles in recycling partially degraded tRNAs and maintaining a population of functional tRNAs, possibly by a constitutive activity on all tRNAs. Proving the existence of this activity *in vivo* is challenging, because the nucleotide before and after editing remains the same unless a G–U base pair is edited to a canonical Watson–Crick base pair. Finally, it is important to consider that the possibility that a cooperation between 5'- and 3'-editing, although not yet observed to occur simultaneously in any system, could also exist in order to deal with degradation or other damage to 5'- and 3'-ends.

### 5.4. Apparent Absence of Editing outside of Eukaryotic Organelles

It remains a mystery why both 5'- and 3'-editing are observed in eukaryotic mitochondria and chloroplasts, which are generally considered to have evolved from bacterial ancestors, while no corresponding events have been identified in bacteria or archaea. For 5'-editing, the possibility of using bacterial and archaeal orthologs of the 3'–5'-polymerases in tRNA repair pathways (whether as part of programmed editing of some unidentified type or as part of more general surveillance systems) is an attractive idea, but evidence for bona fide cellular functions for these Thg1/TLP orthologs remains to be obtained. A scenario can be envisioned where bacterial and archaeal cells could be completely intolerant to mutations that generate mismatches on tRNA because of the inability (or absence) of appropriate nucleases to cleave the mismatched nucleotides and thus generate appropriate substrates for the 3'–5' polymerases.

Moreover, 5'- and 3'-editing has not been associated with any nuclear-encoded tRNAs in Eukarya, except for the highly specialized G-1 editing reaction for tRNA^His^. Nonetheless, in *S. cerevisiae*, the TRAMP complex exists outside the mitochondrion and can act as a 3'-editing enzyme. Moreover, some eukaryotic 3'–5'-polymerases of the Thg1/TLP family are predicted to localize to the cytosol of *D. discoideum* and *A. castellanii*, yet no substrates for the enzymes have been identified in these compartments. Together, these observations suggest the potential for 5'- and/or 3'-editing-like activities in the cytosol or nucleus that may yet be identified.

Whether or not there is an intrinsic value to any type of tRNA editing, as opposed to directly encoding complete and intact tRNA sequences, remains an open question that is beyond the scope of this review. Nonetheless, the previous existence of enzymes that are capable of catalyzing the required changes to tRNA sequences is clearly a prerequisite for the ability to tolerate variations in tRNA sequence that could have led to the evolution of these pathways [6], and it is intriguing that both of the enzymes so far associated with 5'- and 3'-editing (the TRAMP complex and TLPs) are members of larger and more widespread enzyme superfamilies with roles beyond tRNA editing. Perhaps, under the pressures that led to significant compaction of the mitochondrial and chloroplast genomes, this led to a need for more examples of polycistronic transcripts and overlapping tRNA, which in turn led to the currently observed patterns of tRNA editing reactions.
